# Outcomes of Percutaneous Cholecystostomy in the Management of Acute Cholecystitis: A Retrospective Cohort Study at a Rural District General Hospital

**DOI:** 10.7759/cureus.95838

**Published:** 2025-10-31

**Authors:** Michael Rimicans, Joshua Fultang, Luke Thompson, Krishna Oochit, Karthik Maruthachalam

**Affiliations:** 1 General Surgery, Dumfries & Galloway Royal Infirmary, Dumfries, GBR

**Keywords:** acute cholecystitis, comorbid patient management, general surgery, percutaneous cholecystostomy, retrospective cohort study

## Abstract

Background

Percutaneous cholecystostomy is a recognized option for managing acute cholecystitis in high-risk surgical patients unsuitable for laparoscopic cholecystectomy. Tools such as the American Society of Anesthesiologists (ASA) grading and the Charlson Comorbidity Index (CCI) scoring are often used to suggest which patients would have better outcomes with percutaneous cholecystostomy. According to the literature, recurrence rates and leukocytosis reduction are key measures of cholecystostomy's clinical effectiveness in managing cholecystitis.

Aims

This study sought to evaluate clinical outcomes after percutaneous cholecystostomy in a rural district general hospital, specifically comparing outcomes for patients identified as being the best candidates for Percutaneous cholecystostomy (PC) according to the Tokyo Guidelines with outcomes for patients who received PC despite not being adherent to the criteria of the Tokyo Guidelines.

Methods

This was a retrospective cohort study. Sixty patients underwent PC for acute cholecystitis in a single rural district general hospital between October 2019 and January 2024. Data collection was carried out on clinical information systems to gather relevant variables such as patient demographics, blood results, comorbidities, complications, and length of stay in the hospital.

Results

Sixty patients underwent cholecystostomy during the study, with data available for 58 (96.7%). Of these, 21 (36.2%) were female. The mean age was 74±10.5. The average ASA was 2.9, and the average CCI was 6.0. Of all patients who underwent a cholecystostomy, there was a technical success rate of 100%. Of the 48 (82.8%) patients with a leukocytosis on the day of procedure, 48% (n=21) experienced resolution of leukocytosis 48-72 hours postoperatively. The average reduction in white cell count from the day of procedure to 48-72 hours post-operatively was 36.8% (p= 0.002 × 10^-5^). There was a non-fatal complication rate of 31% (n=18) and the 30-day mortality rate was 5.1% (n=3). Of the patients who underwent PC, 35% (n=20) later went on to receive laparoscopic cholecystectomy.

Conclusion

PC has been shown to be an effective procedure in the management of acute cholecystitis in patients identified as high-risk using ASA grading and CCI scoring.

## Introduction

Background

An estimated prevalence of gallstones of 6% of the general population leads to a significant proportion of patients presenting to the general surgical department as an emergency with gallstones and related complications [1-3]. According to Rubio-García et al., 30% of acute admissions to general surgery are due to acute cholecystitis (AC) [4]. Current evidence suggests that laparoscopic cholecystectomy (LC) remains the gold standard treatment for acute cholecystitis [2]. Percutaneous cholecystostomy (PC) is an alternative procedure to LC in the management of AC for patients who are unsuitable for cholecystectomy, either due to the severity of presentation and/or medical comorbidity [5,6]. This is supported by the 2018 Tokyo Guidelines (TG), which aim to standardize the management of patients with suspected or confirmed AC based on illness severity and patient risk due to comorbidities, as measured by the American Society of Anaesthesiologists (ASA) grade and Charlson Comorbidity Index (CCI) scoring systems, if they were to undergo LC [7]. The goals of treatment in the management of AC are resuscitation, removal of foci of infection, and treatment with broad-spectrum antibiotics [2,7]. PC is intended to achieve local source control of infection in patients deemed to be too high risk for LC, and the TG proposes that these patients might be those with an ASA grade ≥3 and CCI ≥6 [5].

PC is normally performed via a transhepatic approach using a Seldinger technique and carries a high technical success rate of 97-100% with the Society of Interventional Radiology having established a technical success threshold target of 97.9% [8]. In the same publication, the society also defines clinical success as resolution of sepsis or a significant reduction in patient group mortality, although it is also recognized that patients undergoing PC often die due to comorbidities rather than as a direct consequence of PC or AC.

Aims

This study sought to evaluate clinical outcomes after percutaneous cholecystostomy in a rural district general hospital, specifically comparing outcomes for patients identified as being the best candidates for PC according to the TG with outcomes for patients who received PC despite not being adherent to the criteria of the TG.

## Materials and methods

A retrospective data collection was carried out for all patients who received PC during the period October 2019 - January 2024 at a single rural district general hospital. A local interventional radiology database held for all radiologically inserted abdominal drains was screened to identify PCs inserted to manage AC during the study period. Pericholecystic collection and liver abscess drains were excluded from the study so that only those who underwent PC following a diagnosis of PC (made from combined clinical, biochemical, and radiological findings) were included. Cases where there were incomplete medical records data were also excluded (n=2).

Variables of interest included ASA grade, CCI score, patient demographics, namely, age, sex, functional status, and pre- and post-insertion C-reactive protein (CRP) and white cell count (WCC) levels. Key outcome measures included response of inflammatory markers, 30-day and 1-year mortality rates, length of hospital stay, intensive care unit (ICU) admission rates, complication rates from PC insertion, duration of drain, the number of patients who subsequently underwent cholecystectomy, and their associated complication rates. All of this data was manually extracted from the local clinical information systems before statistical analysis was performed using Microsoft Excel software, which included calculation of the paired t-test for WCCs prior to PC and 48-72 hours post PC.

## Results

Sixty patients underwent a PC within the timeframe of the study. Of these, adequate data were available for 58 (96.7%) patients. Of these, 31 (53.5%) were male, and the mean age was 74±10.5 years, as seen in Table 1.

**Table 1:** Demographics of patients who underwent PC CRP: C-reactive protein (Units); WCC: White cell count (10×10^9^/L)

Sex M/F (Number)		Male 31	Female 27		
	N	Mean	SD	Range	
Age	-	74	10.5	56-94	
CRP on admission	-	189	132	4-489	P-value 0.012
CRP on day of PC	-	249	129	15-562	
WCC on admission	-	16	6	6 - 33	P-value 0.79
WCC on day of PC	-	17.4	6.6	6.0-30	
WCC 48-72 hours after PC	-	11.0	6.7	4.4 – 46.6	-
Change in WCC 48-72 hours after PC compared to day of PC	-	6.5	6.9	+16.2 –(-20.9)	P-value 0.002 × 10^-5^
Number of cases with leukocytosis (≥10.00 × 10^9^/L) on day of PC	48	-	-	-	-
Number of cases with resolved leukocytosis 48-72 hours after PC	21	-	-	-	-

**Figure 1:**
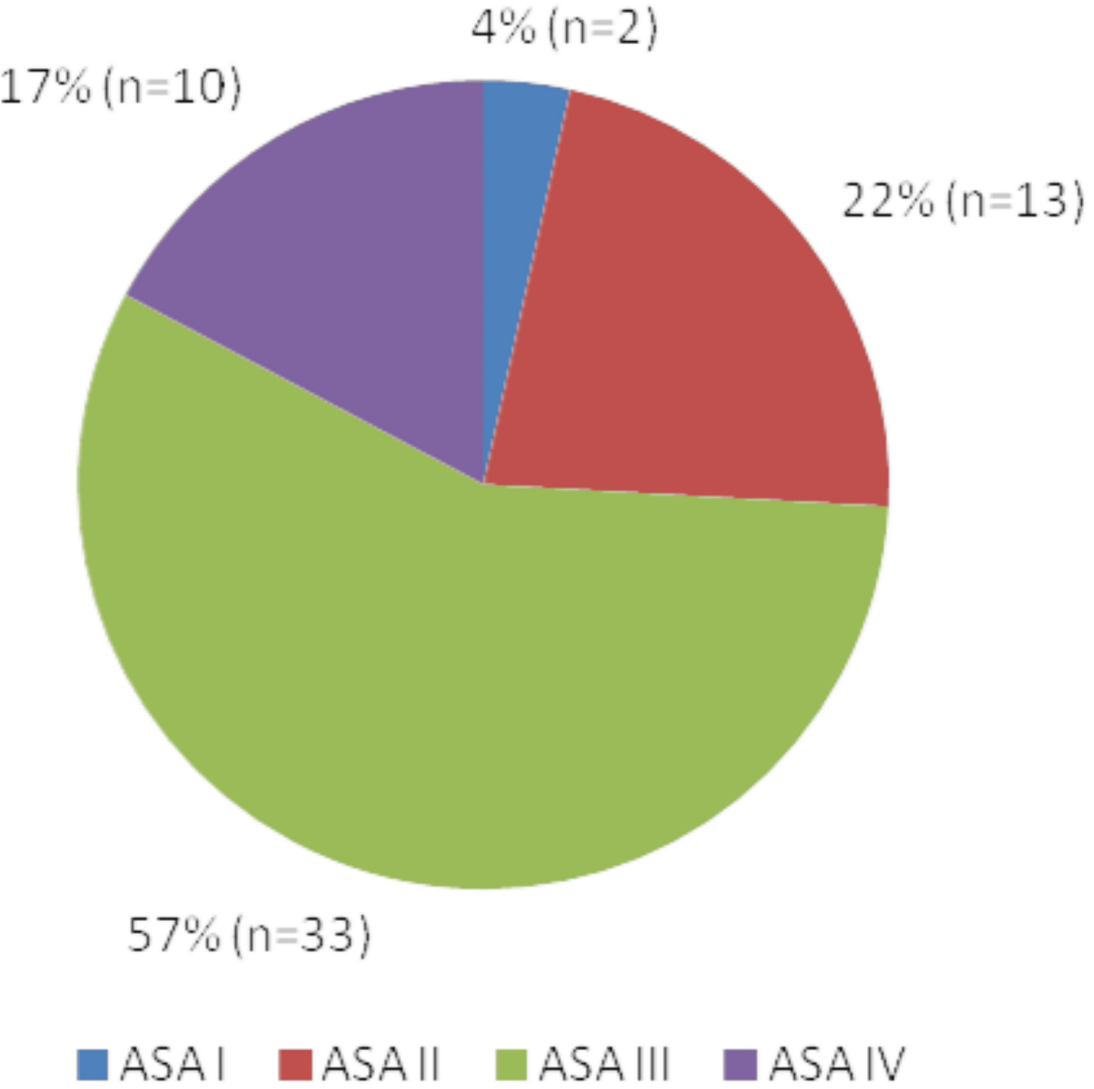
Distribution of ASA Grades of Patients who Underwent PC ASA: American Society of Anesthesiologists; PC: Percutaneous cholecystostomy

**Figure 2:**
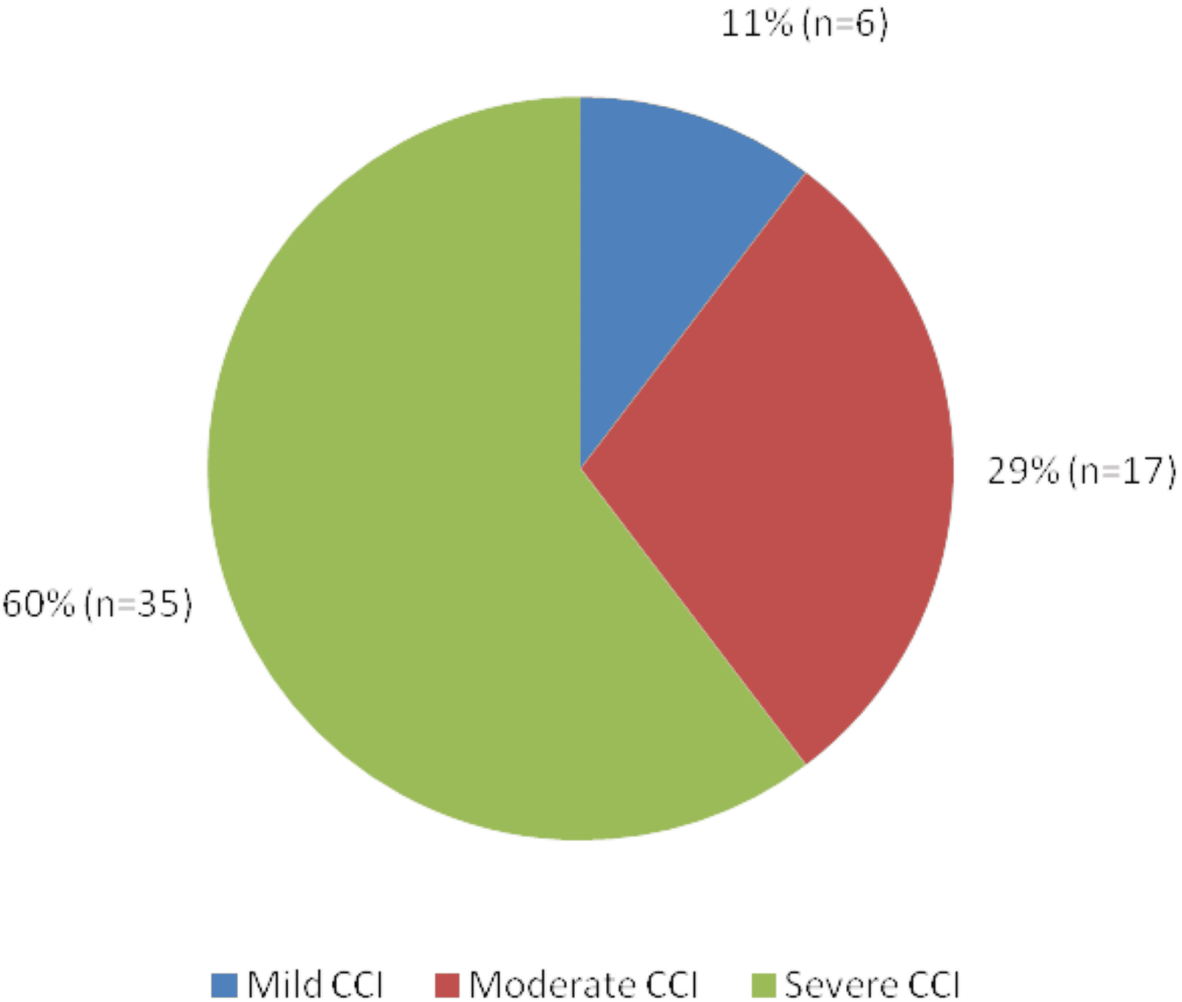
Distributions of Grading of Charlson Comorbidity Index Scores of Patients who Underwent PC PC: Percutaneous cholecystostomy

As seen in Figure 1 below, 73% (n=42) of patients were ASA Grade III or above, 29% (n=17) had a moderate CCI (3-4), and 60% (n=35) had a severe CCI score of 5 or above (Figure 2).

All PCs were inserted using a transhepatic approach using ultrasound guidance. There was a technical success rate of PC of 100% (n=58). The PC was in situ for an average duration of 48±49 days; 19% (n=11) of whom had their PC in for > 80 days. Of all patients who underwent a PC, 15.5% were admitted to the critical care unit (n=9).

Cholecystograms prior to removal of PC were carried out in 46.5% (n=26) of patients, of which 23% (n=6) were abnormal, as seen in Table 2. 

**Table 2:** Cholecystogram Results

	Total PCs (n)	Cholecystogram Performed (n)	Cholecystogram not Performed (n)
Total number of cases	58	26	32
Normal cholecystograms		20	
Abnormal cholecystograms		6	
Persistent gallstones		1	
CBD stones		1	
Cystic duct stones		1	
Blocked cystic duct		1	
Contrast leaks of unknown etiology		2	

The mean length of hospital stay (LOHS) was 19±26 days. Complication rates from the PC insertion procedure were 6.9% (n=4). As seen in Table 3, the 30-day and 1-year mortality rates were 5.1% (n=3) and 15.5% (n=9), respectively. The mean number of care episodes following a discharge with the PC in situ was 2±1.5. Of all 58 patients who were managed with PC, 35% (n=20) had a subsequent cholecystectomy, of which 10% (n=2) were converted to open from laparoscopic.

**Table 3:** Outcomes of percutaneous cholecystostomy

	PC Meeting TG Criteria		PC not Meeting TG Criteria		P-value	All PC Combined		
						Number	%	
	Number	%	Number	%				
30-days mortality	3	6.8	0	0	0.56	3	5.1	
1-year mortality	7	16	2	14	1.00	9	15.5	
Subsequent cholecystectomy	14	32	6	43	0.67	20	35	
Non-fatal complications	17	41	1	7	0.03	18	31	
ICU admission	7	16	2	14	1.00	9	15.5	

The post-operative non-fatal complication rate from PC was 31% (n=18) which included dislodged PC (n=9, 16%), PC forcibly removed by patient (n=1, 2%), blocked PC (n=2, 4%), bile bypassing drain (n=1, 2%), bile leak into abdominal cavity (n=1, 2%), PC site infection (n=3, 5%), abdominal wall abscess requiring incision and drainage (n=1, 2%), sepsis attributed to PC (n=1, 2%) and severe right upper quadrant abdominal pain attributed to PC (n=6, 10%).

## Discussion

As suggested by the literature, our data show that a PC is a safe and effective way of managing AC in selected patients. A recent systematic review by Karakas et al. recognizes that while resolution of sepsis and reduction in mortality rates are prudent goals of treatment in PC, as recognized by the Society of Interventional Radiology, the comorbidity of the patient group typically undergoing PC necessitates a clearer measure of PC effectiveness [5,8]. They propose that a reduction in WCC post PC is the gold standard objective measure of success of PC, and earlier research from other authors has correlated this with good clinical outcomes [9-13]. We found that there was no significant difference in inflammatory markers (WCC and CRP) from the day of admission to the day of cholecystostomy (p=0.43), reflecting a failure to respond to initial conservative management with antibiotic treatment. Of the 48 patients who had pre-PC leukocytosis (defined as WCC ≥10.0 × 109/L), 44% (n=21) were shown to have resolution of leukocytosis 48-72 hours post PC. The average WCC on the day of PC was reduced by 36.8% 48-72 hours post PC.

Adherence to the TG for the 5-year period studied was 76%. Although patients with lower ASA and CCI scores who received PC despite being under the TG threshold had zero observed complications and a lower 30-day and 1-year mortality, a higher percentage of these patients, compared to those meeting the TG for PC, went on to eventually receive LC. The obvious question to ask in a clinical setting would be whether these patients should have had an LC rather than a PC, given the observed increased rate of laparoscopic converted to open procedures in these patients who subsequently had a cholecystectomy. The recent CHOCOLATE trial found that patients with lower ASA grades who were non-adherent to the TG who underwent PC had higher mortality and readmission rates and longer LOHS compared to those of the same ASA grade managed with early LC [14]. While PC does not provide definite management of gallstone pathology as with LC, this could be considered permissible in a comorbid patient group with a shorter life expectancy, compared to the general population, where there is expected to be a lower risk of recurrence of AC within their lifetimes.

There are limitations to this retrospective cohort study. Exclusion of cases with incomplete medical records meant there was a selection bias. For patients who did not meet the TG criteria for PC but still underwent PC, the decision to offer PC was the result of a holistic clinical assessment rather than clearly defined and categorizable justifications.

Quality of life and cost-effectiveness of PC versus LC are not a consideration of the TG or the wider literature so far, and this information could further enhance decision-making with regard to the management of high-risk patients with AC.

## Conclusions

Overall, PC has been shown to be a reasonable treatment option for high-risk surgical patients with AC, where a reduction in WCC has provided objective evidence of clinical effectiveness and there is a low rate of complications, mortality, and recurrence, particularly for the majority of patients in this study for whom their treatment with PC was adherent to the TG. For those who underwent PC without adherence to the TG, there is some evidence to suggest that these patients would have had better outcomes with early LC.

Future research could consider the impact on quality of life and cost-effectiveness of PC compared to alternative management for AC in high surgical risk patient groups.
